# CT引导射频消融与瘤内化疗治疗早期非小细胞肺癌的临床研究

**DOI:** 10.3779/j.issn.1009-3419.2016.05.04

**Published:** 2016-05-20

**Authors:** 威健 冯, 进 李, 素红 韩, 金峰 唐, 洁 要, 玉清 崔, 春堂 王, 忠诚 陈, 晓光 李, 修益 支

**Affiliations:** 1 100038 北京，首都医科大学附属复兴医院，首都医科大学肿瘤学系 Department of Oncology, Fuxing Hospital Affiliated to Capital Medical University, Beijing 100038, China; 2 050011 石家庄，河北医科大学附属石家庄平安医院 Department of Oncology, Shijiazhuang Pingan Hospital Affiliated to Hebei Medical University, Shijiazhuang 050011, China; 3 100054 北京，北京健宫医院 Department of Oncology, Beijing Jiangong Hospital, Beijing 100054, China; 4 100045 北京，武警北京总队第二医院 Department of Thoracic Surgery, Beijing Wujing No.2 Hospital, Beijing 100045, China; 5 253000 德州，德州市肿瘤医院 Department of Thoracic Surgery, Dezhou Cancer Hospital, Dezhou 253000, China; 6 137000 白城，白城市立医院 Department of Oncology, Baicheng City Hospital, Baicheng 137000, China; 7 100005 北京，北京协和医院 Department of Interventional Radiology, Beijing Union Hospital, Beijing 100005, China; 8 100054 北京，首都医科大学宣武医院 Department of Thoracic Surgery, Beijing Xuanwu Hospital of Capital Medical University, Beijing 100054, China

**Keywords:** 肺肿瘤, 射频消融, 瘤内化疗, Lung neoplasms, Radiofrequency ablation, Intratumor chemotherapy

## Abstract

**背景与目的:**

射频消融（radiofrequency ablation, RFA）已经成为无法手术的早期非小细胞肺癌（non-small cell lung cancer, NSCLC）的局部治疗方法之一。本研究观察计算机断层扫描（computed tomography, CT）引导下RFA与瘤内化疗（intratumoral chemotherapy, ITC）（RFA-ITC）的有效性和安全性。

**方法:**

自2005年1月至2015年12月研究组前瞻性入组经病理学证实为早期NSCLC，因心肺功能较差或伴发其他疾病而无法耐受手术或拒绝手术的患者，接受RFA-ITC治疗。RFA采用导向器辅助CT引导穿刺准实时步进法，适形伞状电极、单点或多点消融，完成治疗计划并当CT显示肿瘤周围正常肺组织呈现磨玻璃样后结束消融治疗，经电极针将卡铂200 mg缓慢注射到肿瘤内。随访评估安全性和有效性。

**结果:**

110例患者125次RFA-ITC治疗，技术成功率为100%。中位生存期为48.0个月，总生存率为55.4个月，无进展生存期为55.1个月；1年、2年、3年、5年总生存率分别为100%、90.7%、62.7%、21.9%。消融后有和无磨玻璃样改变的生存期分别是68.3个月、40.1个月，有统计学差异（*P*=0.001）。肿瘤的大小及有无N1分期的生存率无差异。无围手术期死亡发生，气胸、肺内出血、胸腔积液、发热、术中胸痛、皮下气肿、术中咳嗽等并发症轻微可耐受。

**结论:**

CT引导RFA-ITC治疗不能手术的早期NSCLC，疗效好、并发症少，对患者损伤小，为不能手术治疗的早期NSCLC的治疗提供了一个良好方法。

## Abstract (en)

**Background and objective:**

Radiofrequency ablation (RFA) has become one of the local treatment for inoperable early stage non-small cell lung cancer (NSCLC). This study observes effectiveness and safety of computed tomography (CT) guided RFA followed intratumoral chemotherapy (RFA-ITC).

**Methods:**

From 2005 to 2015, our group perspectively enrolled inoperable early stage NSCLC underwent RFA-ITC duo to poor cardiopulmonary function or with other diseases or patient can't tolerate or reject surgery. RFA was performed by a directive apparatus assisted CT guided semi real-time and step-by-step puncture method, conformal umbrella-shaped electrode and single or multiple targets ablation. While the plan finished and CT showed normal lung tissue around the tumor present ground-glass opacity (GGO), the procedure ended, then 200 mg of carboplatinum were injected into the tumor via the electrode needle. Safety and effectiveness were evaluated by follow-up.

**Results:**

Technical success rates of 125 RFA-ITC treatments of 110 patients were 100%. The median survival was 48.0 months, overall survival (OS) was 55.4 months, progression-free survival was 55.1 months, 1, 2, 3, 5-year OS rates were 100%, 90.7%, 62.7%, 21.9%, respectively. Survival of GGO presence or not was 68.3 months and 40.1 months, respectively (*P*=0.001). The survival rates of the N1 staging and tumor size was no significant difference. No perioperative deaths occurred, the main complications i.e. pneumothorax, pulmonary hemorrhage, pleural effusion, fever, intraoperative chest pain, subcutaneous emphysema, intraoperative cough were slight and tolerable.

**Conclusion:**

CT guided RFA-ITC provides a good method for treatment of inoperable early stage NSCLC with better survival, less complication and small damage.

肺癌已经成为最常见和致死率最高的恶性肿瘤^[1]^，非小细胞肺癌（non-small cell lung cancer, NSCLC）采用分期多学科综合治疗，早期NSCLC首选手术切除。但是，部分患者因心肺功能不全或伴发其他疾病而无法耐受手术。射频消融治疗（radiofrequency ablation, RFA）已经成为无法接受手术的早期NSCLC患者的可选择治疗方法之一^[2]^。本课题组自2005年以来开展了CT引导下RFA与瘤内注射化疗药物（intratumoral chemotherapy, ITC）联合应用（RFA-ITC），现对其有效性和安全性报告如下。

## 1 资料和方法

### 1.1 病例选择

纳入2005年1月-2015年12月，课题组所在医院肿瘤科、介入科和胸外科对经病理学证实为NSCLC，影像学或者正电子发射型计算机断层显像（positron emission tomography-computed tomography, PET-CT）等临床肿瘤-淋巴结-转移（tumor-node-metastasis, TNM）分期属于早期（Ⅰ期、部分患者为Ⅱ期），因心功能不全、肺功能较差或伴发其他疾病而无法耐受手术、或拒绝手术的患者，在获得知情同意后入院实施CT引导下的RFA-ITC治疗。排除标准：东部肿瘤合作组（Eastern Cooperative Oncology Group, ECOG）评分≥2分，肿瘤距离重要器官如主支气管、大动脉、心脏和食管等≤1 cm，伴有严重的感染、凝血障碍等。

### 1.2 治疗前准备

术前常规及分期影像检查。禁食水2 h以上，训练呼吸于呼气末闭气。给予镇静、止血、止痛、镇咳预处理。根据病灶位置选择治疗时的体位，经皮穿刺的通路以避开肋骨、血管、减少穿刺胸膜、距离短而安全为目标。术中持续吸氧，监测血压、心电、血氧和脉搏。

### 1.3 CT引导穿刺

CT为GE、Siemens等多种型号，常规低剂量5 mm层厚扫描。穿刺部位体表放置定位尺和支撑支架，穿刺通路为病灶最大层面体表进针点和靶点之间连线，测量深度和角度。CT引导穿刺在局部麻醉和无菌操作下，采用导向器辅助下的准实时步进法，呼气末闭气下进行。导向器的双向角度分别与CT机架角度和穿刺进针角度一致。使CT激光定位线与穿刺针完全重合（图 1）。CT扫描可见穿刺针及尾影通过靶点的中心。采用一步法或者分步法，将穿刺针插入相应深度命中靶点。

**1:**
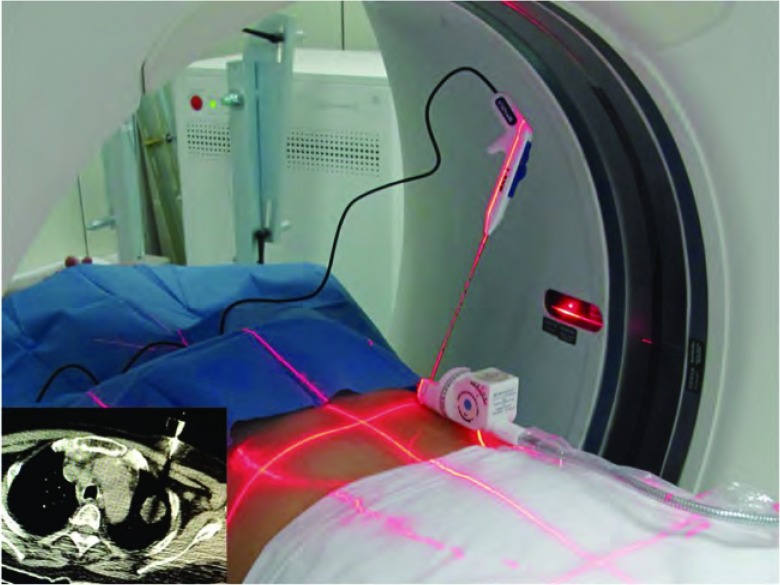
导向器辅助的CT引导穿刺：将CT激光定位线与导向器支撑的穿刺针完全重合，CT扫描可见穿刺针的延长线或者尾影通过靶点（左下图），迅速穿刺至相应深度，即可命中靶点 A directive apparatus assisted CT guided puncture method: overlap the CT laser light with puncture needle, CT scan shows an extension cord or shadow tail of needle through the target (lower left), then puncture to the depths rapidly to targeting the lesion. CT: computed tomography

### 1.4 射频消融

射频消融仪为WE7568型（北京为尔福电子公司），频率290 KHz，最大输出功率为300 W，自动控温（90.0±0.5）℃。消融电极针为WHK适形伞状电极，针尖为炮弹型，主针尖端有实时测温电偶，12支子针可分为两组分别释放至50 mm、可回钩成锚状，并带有注药和针尖测温功能（图 2）。射频治疗计划如表 1所示，通常自肿瘤中心开始（当肿瘤较大时，可以多靶点，此时消融从穿刺的近端靶点开始），设置温度为90 ℃，自动脉冲功率逐渐提升，消融治疗时间，根据肿瘤的大小制定治疗计划：当靶点升温至90 ℃时开始计时，根据肿瘤的直径，逐步打开子电极针的长度，完成一个靶点的治疗，直至子电极释放达到肿瘤的边缘，治疗靶区（planned tumor volume, PTV）为肿瘤直径（gross tumor volume, GTV）外放10 mm。治疗中复查CT，当显示肿瘤周围5 mm-10 mm的正常肺组织呈现磨玻璃样（ground-glass opacity, GGO）改变，则可以结束消融治疗。

**2:**
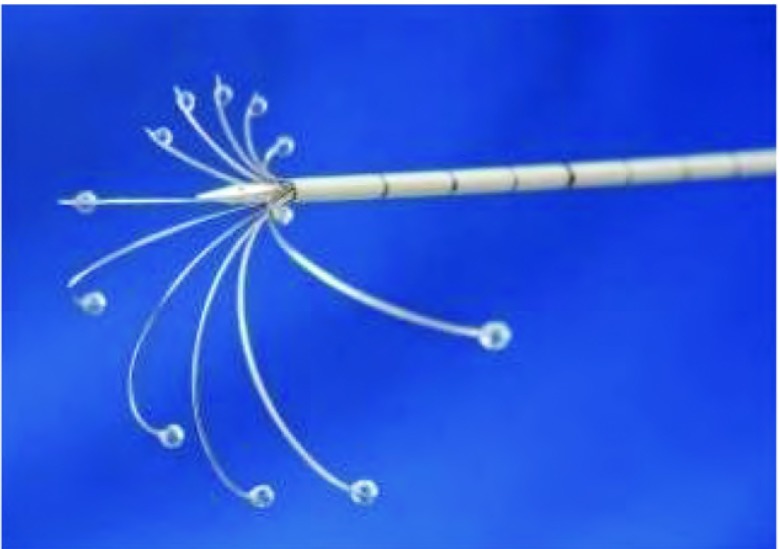
伞状适形射频消融电极针：子针可以两组分别释放并带有注药功能，主针和子针尖端实时测温 Umbrella-shaped conformal RFA electrode: fine needles can release respectively with injection and the real-time temperature measuring at the tip of needles

**1:** 消融计划：肿瘤直径、靶点数量、靶点位置与维持90 ℃所需消融时间（min） Ablation plans: Tumor diameter, number of targets, target position and the ablation time at 90 ℃ (min)

Tumor diameters (mm)	No of target	Target position	Release length of fine electrode needles near to tumor margin (mm)					
			10	20	30	40	50	-
d < 20	Single	Centre	5	10	GGO^*^	-	-	-
20≤d < 30	Single	Centre	5	5	10	GGO^*^	-	-
30≤d < 40	Single	Centre	5	5	5	10	GGO^*^	-
40≤d < 50	double	15 mm interval	5	5	5	5	10	GGO^*^
d≥50	≥Triple	15 mm interval	5	5	5	5	10	GGO*
^*^Planned tumor volume (PTV): While ablation plan completed, the procedure would be finished if changes of normal lung tissues around the tumor present ground-glass opacity (GGO).								

### 1.5 肿瘤内注射化疗

消融结束后，待靶区温度降至60 ℃以下时，经消融电极针将溶于1 mL 5%葡萄糖注射液的卡铂200 mg，通过射频消融电极针的注射孔，一边回收子针一边缓慢注射到肿瘤内。最后回收子电极，针道消融同时拔针，包扎穿刺点。再次CT扫描，观察病灶变化、药物的分布和有无气胸、出血等并发症发生。

### 1.6 术后治疗

明确患者无异常后送返病房，卧床4 h以上，心电监护4 h-8 h。高风险患者（心肺功能差等）预防性使用抗生素。术后24 h行胸部正侧位X线检查，再次明确并发症发生情况。

### 1.7 治疗评估与随访

术后即刻、1个月、1年内每3个月，3年内每年，以后每2年随访。评估影像学随访采用CT、磁共振成像（magnetic resonance imaging, MRI）或PET-CT扫描，统计技术成功率、安全性和有效性。治疗过程评价包括穿刺命中率和治疗成功率：经皮穿刺靶点的命中率，射频消融按计划实施，完成治疗目标，安全性评估穿刺和射频消融相关的并发症，包括术中与术后的生命体征、疼痛评估、出血、气胸、发热等。

局部疗效评估包括：消融范围（GGO形成）：术后即刻GGO形成和24 h GGO变化及1个月GGO的吸收情况；消融率，以射频术后1个月病灶大小为新的基线，术后3个月评价疗效，评价标准采用修订的肿瘤热消融疗效评价标准，分为：完全消融（complete response, CR）：病灶消失，形成空洞，纤维化，疤痕化；实性结节缩小或无变化，但CT无强化、PET-CT为无代谢区。部分消融（partial response, PR）：肿瘤残存，CT扫描有部分强化，PET-CT仍有代谢区。局部进展（progressive disease, PD）：病灶及强化区域均增大10 mm以上、PET的活性区域增大或者增加。

远期疗效评估包括：无进展生存期（progression-free survival, PFS）：从术后第1天至靶病灶进展或出现新发病灶及区域淋巴结或远处转移；中位生存时间：从术后第1天至50%病例生存的时间；总生存期（overall survival, OS）：从术后第1天至死亡或最后一次随访。

### 1.8 统计学方法

采用SPSS 17.0统计分析软件，生存分析采用*Kaplan*-*Meier*法，*P* < 0.05为差异有统计学意义。

## 2 结果

### 2.1 基本资料

本研究入组患者110例，男性66例，女性44例，平均年龄67.3岁（30岁-86岁），肿瘤直径平均37 mm（范围15 mm-60 mm）。采用2015版TNM临床分期：Ⅰ期87例（79.1%）[Ⅰa（T1, N0, M0）期，52例（47.3%）；Ⅰb（T2a, N0, M0）期35例（31.8%）]；Ⅱ期23例（20.9%）[Ⅱa（T2b, N0, M0）期14例（30%），（T1, N1, M0）期3例（2.7%），（T2a, N1, M0）期3例（2.7%），Ⅱb（T2b, N1, M0）期3例（2.7%）]。组织病理学类型为：鳞状细胞癌40例（36.4%），腺癌70例（63.6%）。病变部位：右肺61例（右上叶15例，右中叶18例，右下叶28例）；左肺49例（左上叶20例，左下叶29例）（表 2）。入组患者均因高龄、心肺功能差及其他伴发疾病无法耐受手术或拒绝手术，签署知情同意后实施RFA-ITC治疗。

**2:** 基本资料 Patient characteristics

Charictristics	Data
Gender (male/female)	66/44
Age (yr)	
Mean	67.3
Range	30-86
Tumor size (mm)	
Mean	37
Range	15-60
Clinical stage	
Stage Ⅰ	87
Ⅰa (T1, N0, M0)	52
Ⅰb (T2a N0 M0)	35
Stage Ⅱ	23
Ⅱa (T2b, N0 M0)	14
(T1 N1, M0)	3
(T2a, N1, M0)	3
Ⅱb (T2b, N1, M0)	3
Pathohistology	
Squamous carcinoma	40
Adenocarcinoma	70
Lobar location	
RUL/RML/RL	15/18/28
LUL/LLL	20/29
LLL: left lower lobe; LUL: left upper lobe; RLL: right lower lobe; RML: right middle lobe; RUL: right upper lobe.	

### 2.2 治疗过程评价

本研究110例均顺利完成操作治疗，全部病例实现单针道穿刺，穿刺命中率为100%；总计完成125次CT引导RFA-ITC治疗，1次102例，2次9例，5次1例，技术成功率为100%。

### 2.3 安全性评价

本组未发生30日内围手术期死亡，主要并发症为气胸，发生率为4.0%（5/125），其中肺压缩超过30%、且因肺功能较差需胸管引流2例，胸管置入率1.7%；肺内出血4.8%（6/125），CT表现为沿穿刺针道形成的片状阴影，其中2例（1.7%）咯少量血痰，给予止血治疗好转；胸腔积液4.0%（5/125），无需胸管引流。次要并发症包括发热>38.5 ℃ 15例；疼痛是局麻下射频消融治疗的主要并发症，通过降低治疗功率，疼痛可以缓解，之后可逐渐增加功率至可耐受，术中胸痛12%（15/125），降低功率后可以缓解，再次开始治疗疼痛减轻，可以耐受，术后疼痛36.0%（45/125）；皮下气肿2例；呕吐5例，考虑与局部化疗或者注射止痛药有关，予以对症处理。术中咳嗽15例（13.6%），均出现在靶点距离支气管较近（< 5 mm）的病例，暂停治疗，CT复查均未出现异常，再行治疗不再咳嗽。并发症发生与处理情况见表 3。

**3:** 并发症与处理 Complications and managements

Complications	Data	Managements
Pneumothorax	5 (4.0%)	Tube drainage
Tube drainage	2 (1.7%)	Tube drainage
Pneumorrhagia	6 (4.8%)	Hemostatic drug
Hemosputum	2 (1.7%)	Hemostatic drug
Hydrothorax	5 (4.0%)	Tube drainage
Tube drainage	0 (0)	Tube drainage
Fever >38.5 ℃	10 (8.9%)	Indometacin
Thoracodynia during operation	15 (12.0%)	Reduce power
Thoracodynia post operation	45 (36.0%)	Self-limited
Pneumoderm	2 (1.6%)	Analgesics and self-limited
Vomit	5 (4.1%)	Antemetic, self-limited
Cough	15 (13.6%)	Pause power and self-limited

### 2.4 局部疗效评价

治疗后局部GGO的形成和变化：125次治疗中，术后GGO形成为101次（80.8%），其中，肿瘤≤30 mm的55例中，GGO形成49例（89.1%）；肿瘤31 mm-50 mm的38例中，GGO形成30例（78.9%）；肿瘤51 mm-70 mm的19例中，GGO形成7例（36.8%）。通常，24 h后GGO增大5 mm-10 mm，1个月后，GGO消失包绕病灶形成膜样结构，CT平扫治疗区域肿瘤密度降低、无强化，有稍高密度化疗药物影，整体外形较治疗前略大。6个月后，治疗区域呈低密度改变，增强扫描无强化，消融区域逐渐缩小、肿瘤部位形成空洞直至消失。PET/CT高代谢灶消失。治疗后穿刺活检病理学检查为坏死组织，治疗区域玻璃样变、纤维化斑痕形成，伴少量炎细胞浸润（图 3，图 4）。

**3:**
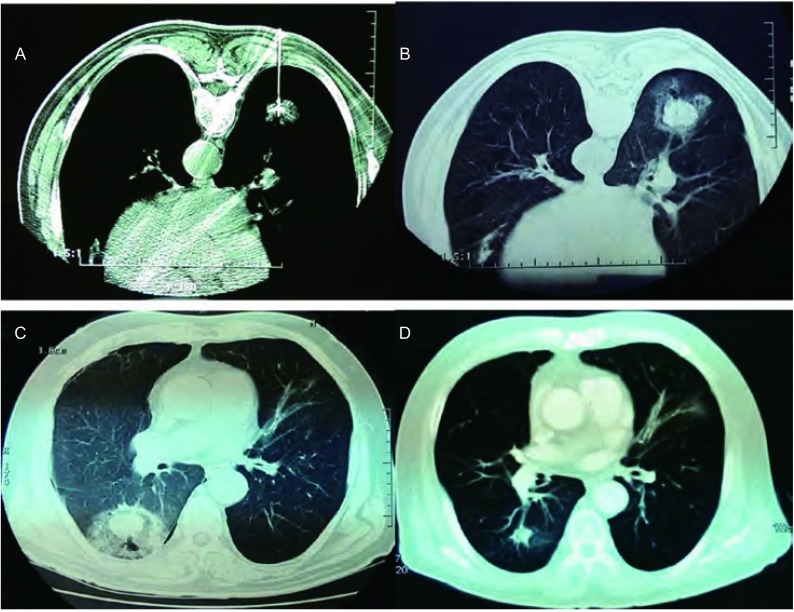
肿瘤的射频消融及周围的肺组织GGO形成的变化过程：A：射频消融；B：手术结束即刻；C：术后24 h；D：术后12个月 Post RFA changes of tumor and GGO presence of normal lung tissues around the tumor. A: RFA; B: immediate; C: 24 h; D: 12 months

**4:**
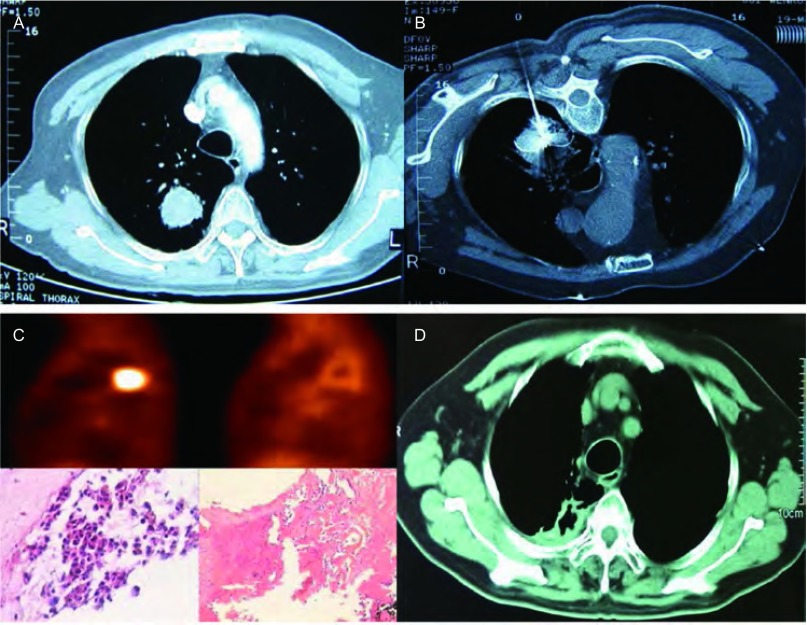
男性80岁肺腺癌（A）患者，射频消融（B）术后复查近血管处仍有残留，追加第2次射频消融，3个月后复查PET肿瘤代谢消失，活检无肿瘤细胞（C），5年后肿瘤消失（D），局部形成瘢痕化空腔，至今存活10年 A 80-year-old male patient with lung adenocarcinoma (A), after first RFA (B), a residual was found near blood vessels, than second additional RFA was performed. PET shows disappearance of tumor metabolism and no tumor cells by biopsy 3 months passed (C), tumors disappeared and shrinked into a small local scarring cavity after five years (D), now the patient has surviving over 10 years

治疗后1个月复查强化CT，完全消融率为89.1%（98/110），部分消融为10.9%（12/110），无疾病进展。其中，肿瘤≤3 cm（Ⅰa期）55例中，完全消融54例（98.2%）；肿瘤3.1 cm-5 cm（Ⅰb期）38例中，完全消融34例（89.5%）；肿瘤5.1 cm-7 cm（Ⅱ期）19例中，完全消融9例（47.3%）。

### 2.5 远期疗效评价

末次随访时间是2015年12月31日，中位随访时间为36个月（范围2个月-120个月），3例失去随访，随访率97.3%，研究结束时，62例生存。110例1年内全部生存，98例生存超过1年，77例生存超过2年，53例生存超过3年，15例生存超过5年，2例生存超过10年（图 4）。

全组中位生存期为48.0个月（范围：44.8个月-51.2个月）。总生存期（overall survival, OS）为55.4个月（范围：46.9个月-63.9个月）；无进展生存期（disease-free survival, DFS）为55.1个月（范围：44.6个月-65.5个月）。1年、2年、3年、5年总生存率分别为100%、90.7%、62.7%、21.9%（图 5A）。有30例（27.2%）于RFA-ITC治疗后约15个月（范围：4个月-24个月）后发生局部复发或者远处转移，10例于复发后接受了再次RFA-ITC治疗。1例T2bN1的鳞癌患者共接受5次射频消融治疗，至今生存达到近5年。另有5例接受放射治疗，10例接受化学治疗。39例死亡，其中20例均死于肿瘤的远处转移，19例死于其他原因如肺部感染、心脑血管疾病。

**5:**
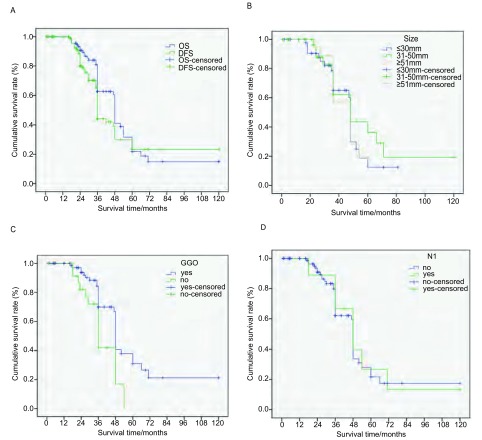
生存曲线。A：无进展生存与总生存曲线（55.1个月*vs* 55.4个月）；B：肿瘤≤30 mm、31 mm-50 mm、≥51 mm的平均生存期的比较（46.7个月*vs* 59.8个月*vs* 44.4个月，*P*=0.711）；C：射频消融后磨玻璃样改变的生存期的比较（68.3个月*vs* 40.1个月，*P*=0.001）；D：无N1淋巴结转移和有N1淋巴结转移患者的生存期的比较（65.2个月*vs* 53.8个月，*P*=0.504） *Kaplan*-*Meier* survival curve. A: PSF and OS (59.0 months *vs* 55.4 months); B: Average survival of tumor ≤30 mm, 31 mm-50 mm, ≥51 mm (46.7 months *vs* 59.8 months *vs* 44.4 months, *P*=0.711); C: Ground-glass opacity (GGO) appearance had or not after RFA (68.3 months *vs* 40.1 months, *P*=0.001); D: comparison of survival between lymph node metastasis N1 no or yes (65.2 *vs* 53.8 months, *P*=0.504)

比较肿瘤≤30 mm、31 mm-50 mm、≥51 mm的三组间的生存期分别为46.7个月*vs* 59.8个月*vs* 44.4个月，差异无统计学意义（*P*=0.711）（图 5B）。消融治疗后出现与未出现磨玻璃样改变的生存期的分别是68.3个月*vs* 40.1个月，组间有统计学差异（*P*=0.001）（图 5C）。无N1淋巴结转移和有N1淋巴结转移病例的生存期分别是65.2个月和53.8个月，组间无统计学差异（*P*=0.504）（图 5D）。

## 3 讨论

随着影像技术的不断进步，越来越多的肺癌被早期发现。临床上，肿瘤≤5 cm而淋巴结阴性（cT2aN0M0）的早期肺癌大约占所有新诊断肺癌15%-20%^[3]^。包括微创手术在内的根治性切除为早期肺癌（T2bN1以内的Ⅰ期、Ⅱ期）提供了治愈的机会，5年生存率可以达到57%-85%。对于因年龄和身体因素不适合或者拒绝手术治疗的患者，可以选择微创或者无创非手术局部治疗手段，如经皮射频消融、立体定向放疗等方法^[4, 5]^。对经过合理选择的肺癌患者进行射频消融治疗也可以提高患者的生存率、甚至达到根治性治疗效果^[6]^，对此，美国国立综合癌症网络（National Comprehensive Cancer Network, NCCN）的NSCLC指南及中国肺癌诊疗规范中均已明确。

射频消融主要是在CT引导下经皮将消融电极插入到肿瘤内部，通过射频交变电流震荡组织分子摩擦产热，测温反馈提示由计算机自动控温在60 ℃-90 ℃，高温凝固组织细胞蛋白，从而杀死癌组织细胞。肿瘤的导电率高于周围肺泡，相同时间内肿瘤的温度远远高于周围肺组织，因此射频消融更适合于治疗肺癌。因此，CT引导下的准确穿刺和对治疗过程的监控，消融的热能分布与调控，是保证射频消融疗效和安全性的关键。

CT是肺部病变经皮穿刺和控制治疗过程的最佳影像技术^[7]^，本研究将CT穿刺引导架^[8]^改进为导向器，起到对穿刺针的辅助支撑、二维定向和在体外瞄准靶点的作用。让CT定位激光线与穿刺针重合，穿刺进针的角度与CT测量的角度完全一致，CT扫描到靶点层面时，穿刺针会出现一条尾影直接指向病灶，当穿刺针沿着这条影子穿刺相应的深度时即可以命中靶点。本研究全部病例实现单针道穿刺，穿刺成功率为100%。为了避开肋骨、大血管、肺大泡及跨肺叶等风险因素，可通过倾斜CT机架角度引导穿刺，提高了准确性和安全性。

气胸是射频消融术治疗肺部肿瘤的主要并发症之一，主要由经皮穿刺所致。气胸的发生率为5%-63%^[9]^，大部分可自愈，只有11%需要放置胸腔闭式引流^[10]^。本研究气胸并发症的发生率仅为4.0%，明显低于文献报道，其中肺压缩超过30%且因肺功能较差需胸管引流2例，胸管置入率1.7%。肺内出血4.8%，CT表现为沿穿刺针道的片状阴影，其中1.6%有少量血痰，给予止血治疗好转。

射频消融使用多种消融电极，包括单极、双极或者多极电极。单极电极又分为灌注电极^[11]^、内冷却电极^[12]^、集束电极^[13]^和多子针电极^[14]^。本研究使用的消融电极具备灌注电极、集束电极和多子针电极的特点，具有一针多功能的独特特点：针尖锋利易于穿刺，减少气胸的发生；向肿瘤组织内部释放10支-12支微细的子电极，导向释放和分两组分别适形释放机构可根据肿瘤的大小和特定的方向适形释放和回收；主电极及子电极均装有测温电偶，治疗中实时反馈靶区的温度，中央控温电极，保证温度控制在90 ℃-95 ℃以内，防止汽化、炭化的发生；通过子针向病变内部注射抗肿瘤药物、止痛药、止血药物以及高渗盐水等，调节子针的长短使药物在肿瘤内部均匀分布。这样的消融电极有利于早期肺癌的RFA-ITC治疗。

在消融电极插入到治疗靶位后，根据温度反馈自动调整功率维持治疗温度。按照消融计划，逐步释放消融子电极至肿瘤的边缘，消融子电极对周围肺组织也会产生一定热辐射的效应，对可能存在亚临床病灶也产生一定的消融治疗作用。

肿瘤内注射抗肿瘤药物，特别是热消融配合化疗药物例如阿霉素，可以增加肿瘤破坏作用、增加疗效^[15]^。铂类抗肿瘤药是NSCLC的主要化疗药物。本研究将RFA与肿瘤内化疗相结合，发挥了局部+区域治疗的优势。射频消融后即刻将卡铂注射到肿瘤内部，CT监控观察铂类药物的分布并通过调节子电极的位置，缓慢均匀将药物浸润分布到肿瘤内。本组9例N1淋巴结转移的患者，治疗后6例淋巴结缩小至消失甚至PET-CT的代谢消失。本研究较少发生淋巴结转移与射频消融配合高浓度的化疗药物，不仅发挥局部抗肿瘤作用，能够通过淋巴回流发挥区域抗肿瘤作用。今后需要对照研究其他溶解度更高的抗肿瘤药物如三代铂类的局部注射应用。

本组患者的耐受性较好，安全性较高。治疗过程中患者会感觉局部发热、出汗甚至心率加快等，无需特殊处理。在消融术后3 d-5 d可能会出现吸收热，体温≤38 ℃，无须特殊处理，超过38.9 ℃给予降温、抗感染处理。治疗中常见有局部疼痛，特别是距离胸膜较近的病变更为明显，与热传导及射频电流刺激有关，通过止痛治疗，或者降低功率，疼痛缓解后再开始治疗，一般多可以较好地耐受。血痰与穿刺损伤或治疗后组织炎性反应有关，可自行吸收，或止血药物治疗2 d-5 d后缓解。热消融刺激少数患者出现胸腔积液，可自行吸收或引流。本研究未出现支气管胸腔瘘、肺脓肿等并发症。本组的并发症发生率较低，可能与我们精选优化射频策略尤其重视穿刺方案的选择有关。

RFA治疗早期NSCLC，1年、2年、3年生存率分别达到78%-100%、57%-84%、36%-74%^[16]^。本组NSCLC患者平均年龄为67.3岁，其中超过70岁占了近一半，最高年龄为86岁，均因各种原因无法接受手术，而选择RFA-ITC治疗。本组患者合并严重肺功能损害或严重合并症的患者较多，110例患者中，98例生存超过1年，77例生存超过2年，53例生存超过3年，15例生存超过5年，2例生存超过10年。全组中位生存期为48个月，总生存与无进展生存期为55个月，1年、2年、3年、5年生存率分别为100%、90.7%、62.7%和21.9%。仅从2年生存率90.7%来分析，RFA-ITC治疗早期NSCLC的疗效，并不低于肺叶切除（85%-95%），甚至高于立体放疗和SBRT（65%-75%）^[3]^，说明RFA-ITC疗法不仅具有较好的局部疗效，还具有一定的全身效果。

本组39例死亡，其中20例均死于肿瘤的远处转移，19例死于非肿瘤相关性疾病，对射频消融的总体生存率产生一定的影响。平均治疗后15个月后发生局部复发或者远处转移，10例于复发后接受了2次、3次RFA-ITC治疗。1例肿瘤直径60 mm伴肺门淋巴结转移的鳞癌，共接受5次射频消融治疗，至今生存达到4年。

完全消融是提高射频消融疗效技术关键。本研究在制定消融计划时，在影像学肿瘤边缘外放10 mm作为“安全界限”。当完成预定的消融计划时，CT扫描确认周围肺组织出现磨玻璃样改变，而对周围正常组织器官没有产生热损伤时，可以达到根治性治疗效果。GGO属于消融后周边肺组织充血、水肿、渗出等形成的炎症反应带，是否完全覆盖原有病灶被认为是RFA成功与否的重要判断依据^[17]^。通常GGO在24 h后稍有增大，1个月后GGO开始消失，缩小包绕病灶形成膜样结构，平扫CT靶区阴影稍有增大，与GGO形成有关。本组患者治疗后80%形成GGO，特别是小肿瘤GGO的形成率高于大肿瘤。射频消融出现GGO的病例的生存期更长，疗效优于未出现者。

临床上，影响肺癌RFA疗效的因素主要有肿瘤大小、部位和是否靠近较大的血管等有关。一般认为最大径 < 3.5 cm的病灶，局部控制率好，相反，大的病灶局部复发率高，生存率明显低于小病灶^[18]^。肿瘤直径 < 3 cm的平均生存期为30.5个月，而肿瘤直径超过3 cm时，生存期为14.9个月^[19]^。本组治疗后1个月随访，胸部CT增强扫描显示，完全消融率为78.4%。特别是肿瘤≤3 cm的患者，完全消融率达到98.2%；肿瘤3.1 cm-5 cm的患者是完全消融率也能够达到89.5%；较大肿瘤5.1 cm-7 cm的完全消融为47.3%，三组的平均生存期分别为46.7个月、59.8个月、44.4个月，总体生存率近似，无统计学差异。

综上所述，CT引导经皮射频消融治疗序贯局部瘤内化疗作为微创治疗技术，治疗不能手术的早期肺癌，近期远期疗效好、并发症少，对患者损伤小，生活质量高，且对于局部复发的病例可再次给予消融治疗，为不能手术治疗的早期NSCLC的治疗提供了一个良好方法。
